# Implementation of a decision aid for recognition and correction of volume alterations (Recova^®^) in haemodialysis patients

**DOI:** 10.1080/03009734.2020.1804495

**Published:** 2020-08-27

**Authors:** Jenny Stenberg, Magnus Lindberg, Hans Furuland

**Affiliations:** a Department of Medical Sciences, Uppsala University, Uppsala, Sweden; b Department of Caring Sciences, University of Gävle, Gävle, Sweden; c Department of Public Health and Caring Sciences, Uppsala University, Uppsala, Sweden

**Keywords:** Body composition, body fluids, decision support techniques, electric impedance, prospective studies, renal dialysis, water–electrolyte imbalance

## Abstract

**Background:**

Fluid overload is associated with mortality in haemodialysis patients, and 30% of patients remain fluid-overloaded after dialysis. The aim of this study was to evaluate if implementation of Recova^®^, a decision aid combining clinical assessment with bioimpedance spectroscopy, facilitates individualization of target weight determination and thereby contributes to improved fluid status in maintenance haemodialysis patients.

**Methods:**

The impact of the implementation was measured as the proportion of participants at an adequate target weight at the end of the study, assessed as change in symptoms, hydration status, and N-terminal pro-brain natriuretic peptide (NT-proBNP). Nurses were instructed to use Recova every 2 weeks, and the process of the intervention was measured as frequencies of fluid status assessments, bioimpedance measurements, and target weight adjustments.

**Results:**

Forty-nine patients at two haemodialysis units were enrolled. In participants with fluid overload (*n* = 10), both overhydration and fluid overload symptom score decreased. In fluid-depleted participants (*n* = 20), target weight adjustment frequency and the estimated target weight increased. The post-dialytic negative overhydration was reduced, but NT-proBNP increased.

**Conclusions:**

Implementation of Recova in haemodialysis care increased the monthly frequencies of bioimpedance measurements and target weight adjustments, and it contributed to symptom reduction.

**Trial registration:**

The Uppsala County Council Registry of Clinical Trials: FoU 2019-0001-15.

## Introduction

Haemodialysis is lifesaving, and its purpose is to replace the vital functions of the failing kidneys. It has two primary goals: to remove uraemic toxins and to restore sodium and water homeostasis (1). The use of an estimated ideal body weight, usually referred to as the dry weight or target weight, remains the standard of care for volume management (2), but there is no consensus on its absolute definition, and even small changes in target weight may be clinically important. Clinical examinations are insensitive to subtle volume alterations, and insufficiently prevent treatment-related complications (3).

In recent years, bioimpedance spectroscopy has gained popularity for assessing body composition and fluid status, due to its simplicity and low cost (3,4). The body composition model describes the intra- and extracellular water (ICW, ECW) content of lean and adipose tissue, and excess fluid, expressed as overhydration (OH) in litres (5). Results from randomized trials evaluating the effect of bioimpedance-guided fluid management are promising; the method can improve blood pressure control, hydration status, and arterial stiffness measurements (6,7). However, there are difficulties associated with reduction of excessive fluid volume, including intradialytic hypotension, ischaemia of heart, brain, and gut, loss of residual renal function, and vascular access thrombosis (8,9). It has been emphasized that bioimpedance cannot provide a simple target applicable to all patients (4,10,11), and how it is actually used to guide the complex intervention of setting a desirable target weight has been difficult to capture (12,13).

Fluid management requires a multidisciplinary effort and a combination of clinically and technically derived parameters. At units where nurses are authorized to change target weight, target weights are adjusted more often, and systolic blood pressure pre-dialysis is significantly lower (14). Also, having a protocol specifying how often to assess target weight in most patients is associated with lower all-cause mortality (15). Nevertheless, most haemodialysis units have not agreed on a fluid management policy (14,16).

A decision aid, Recova^®^, which standardizes the process of recording, scoring, and responding to changes in routinely measured physiological parameters and incorporates bioimpedance in target weight determination, has been developed. The purpose of Recova is to allow early recognition and adequate response to fluid status alterations in haemodialysis patients (17).

The aim of this study was to evaluate if implementation of Recova facilitates individualization of target weight determination and thereby contributes to improved fluid status in maintenance haemodialysis patients.

## Materials and methods

This prospective implementation study was designed as a non-randomized, single-blinded label intervention, carried out at two haemodialysis units within one centre. Patients with end-stage renal disease, undergoing maintenance haemodialysis 2–5 times a week, were screened for enrolment. Criteria for inclusion were haemodialysis treatment ≥3 months, age ≥18 years, and ability to understand and speak Swedish. The exclusion criterion was residual renal function large enough that ultrafiltration was not needed.

Based on the participants’ predominant symptoms and on bioimpedance-measured hydration status, four fluid status groups were defined prior to the intervention: A, symptoms of fluid overload but negative OH; B, symptoms of fluid overload and positive OH; C, symptoms of fluid depletion (or absence of symptoms) but positive OH; D, symptoms of fluid depletion (or absence of symptoms) and negative OH. The categorization of study participants and the estimated urgency of need for correction of target weight were not presented to the staff of the clinics.

### Intervention

The three parts of the Recova tool, which has been thoroughly described by Stenberg et al. (17), were presented to the haemodialysis units’ nurses through workshop sessions:

1. A symptom-scoring system systematizing physiological parameters already used in clinical assessment of fluid status (Figure 1).

**Figure 1.**
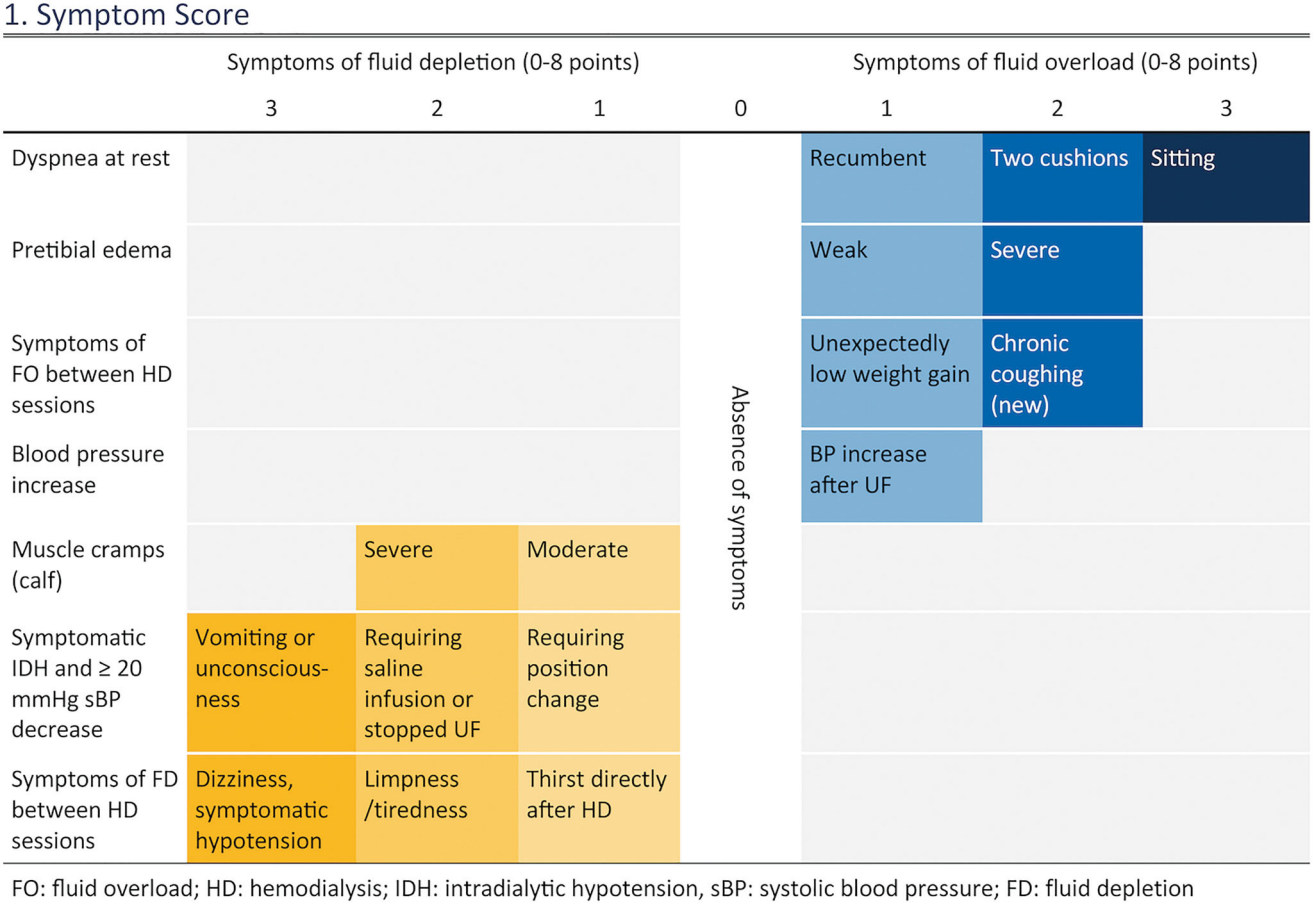
The Recova^®^ scoring system for systemized clinical assessment of fluid status (from Reference [18]). The symptom score can total 0–16 points: 0–8 fluid overload points and 0–8 fluid depletion points.
2. Thresholds and triggers for action indicating any need for action, based on the participant’s total symptom score (Appendix A).
3. A decision aid algorithm, which combines clinical assessment with bioimpedance, and suggests a clinical response and individualized target for OH post-dialysis (OH post) (Appendix B).

The nurses were instructed to use Recova to systematically assess the study participants’ fluid status and to score their symptoms of fluid overload/depletion every 14 days. They were also instructed to respond to the Recova threshold values as appropriate, to perform bioimpedance measurements if necessary, and to alert the nurse or clinician responsible, as recommended in the tool. If appropriate, they were encouraged to initiate target weight adjustments. Target weight determination is the responsibility of the nephrologist, but nurses at most Swedish haemodialysis units are authorized to initiate target weight adjustments of 0.5–1 L (14).

A copy of the complete Recova tool (Figure 1, Appendices A and B) and a protocol for recording assessments were included in the medical record of each study participant. At the morning meetings on the days scheduled for assessment (every 14 days), the nurses on duty were reminded to assess the study participants’ fluid status. In the first haemodialysis unit, the intervention ran for 4 months, May–August 2019. In the second unit, the intervention ran for 3 months, September–November 2019.

### Study of the intervention

The impact of the intervention was measured as proportion of study participants at an adequate target weight at the end of the study, assessed as change in symptoms and hydration status. Also change in N-terminal pro-brain natriuretic peptide (NT-proBNP), a biomarker associated with overhydration in haemodialysis patients (18), was examined. The four fluid status groups, as defined by Recova, the suggested clinical response, and the plausible post-dialysis target weight to aim for in each group are presented in Table 1.

**Table 1.** Fluid status groups, the suggested clinical response and the plausible post-dialysis target weight to aim for in each group – as defined by recova.

	A	B	C	D
Clinically assessed fluid status	Overload	Overload	Depletion or no symptoms	Depletion or no symptoms
OH post (BIS measured)	≤0 L	>0 L	>0 L	≤0 L
Suggested clinical response	Decrease TW 0.5–1 kg/week	Decrease TW 0.5–1 kg/week	First, treat malnutrition and inflammation	Increase TW 0.5–1 kg/week
Plausible OH post target	−2 to 0 L	±1 L	0 to +2 L	±1 L

BIS: bioimpedance spectroscopy; OH: overhydration; TW: target weight.

The process of the intervention was measured as frequency of fluid status assessments and change in frequencies of bioimpedance measurements and target weight adjustments, compared with 6 months prior to the intervention.

### Measures

#### Laboratory data

Blood samples for analysis of NT-proBNP were collected at the beginning of the dialysis session, from the vascular access or from the arterial part of the tubing system, and sent to the hospital’s certified laboratory. Analyses were performed in accordance with the laboratory’s normal routines. Systolic and diastolic blood pressure, body weight, and ultrafiltration volume were recorded. Additional laboratory results, dialysis prescriptions, and retrospective data on frequencies of bioimpedance measurements and target weight adjustments were retrieved from medical records.

#### Measure of hydration status

Each participant’s hydration status was assessed before a mid-week haemodialysis session, at baseline, and after either 3 or 4 months, using the Body Composition Monitor (BCM, Fresenius Medical Care, Bad Homburg, Germany). The BCM measures impedance at 50 frequencies and automatically determines total body water, ECW volume, ICW volume, and OH volume. It defines OH as the difference between the patient’s expected ECW under normal physiological conditions and their actual ECW (19,20). In healthy subjects, normal hydration ranges from −1.1 to +1.1 L OH (21). In this study hydration status was defined as either positive or negative OH, depending on whether a participant’s estimated OH post was >0 L or ≤0 L. OH post was estimated by subtracting planned ultrafiltration volume from OH as measured pre-dialysis.

#### Assessment of fluid status

At baseline, the Recova symptom scoring system was used to clinically assess and score each participant’s fluid status (Figure 1). In order to categorize the participant’s fluid status, depletion score was subtracted from overload score. Depending on whether the sum was positive or negative, fluid status was defined as either fluid overload or fluid depletion.

#### Thresholds for action

In order to measure the urgency of need for correction of the target weight, the total symptom score was calculated. According to Recova, if the total sum is 0, no further action is required, but evaluation of the target weight should be performed every second week. If the score is 1–4, the target weight should be questioned; if it is 5–6, the target weight should be adjusted; and if it is 7 or more, there is an immediate need for evaluation of hydration status and target weight adjustment (Appendix A).

### Statistical analysis

Due to the low sample size, all data were considered non-parametric. Descriptive data are presented as median (Md) and inter-quartile range (IQR) or as percentage/frequency, as appropriate. Differences at baseline between the four groups were tested for significance with Kruskal–Wallis *H* for independent groups of non-parametric variables. Within each group, differences between baseline and end-of-study were tested for significance with Wilcoxon’s non-parametric test for dependent groups. Correlations between measures of hydration status and intervention-driven response were analysed with Spearman’s rank correlation or chi-square tests, as appropriate. Statistical significance was inferred at *P* ≤ 0.05. Statistical analyses were performed using GNU PSPP version 1.2.0, software for statistical analysis (Free Software Foundation, Inc., Boston, MA, USA). For reporting, the SQUIRE guidelines (22), a framework for reporting new knowledge on how to improve health care, were used.

### Ethical considerations

The study complied with the declaration of Helsinki, and all enrolled study participants provided written informed consent. Ethical approval was obtained from the Swedish Ethical Review Authority, Dnr 2019–00011, before the study commenced.

### Trial registration details

The trial was registered in the Uppsala County Council Registry of Clinical Trials (Kansliet för kliniska prövningar); trial registration number: FoU 2019–0001-15, Uppsala, Sweden 2019–02-08. Details available upon request (kliniskaprovningar@akademiska.se).

## Results

### Study cohort characteristics

Forty-nine haemodialysis patients, including 32 males (65%), with a mean age of 73 (67–80) years, were enrolled in the study. The median OH pre-dialysis (OH pre) in the sample was 1.7 (0.9–3.4) L, OH post was 0.10 (−0.80 to 1.3) L, and NT-proBNP was 9270 (2490–19,600) ng/L. Except for OH, fluid overload score, and NT-proBNP, there were no statistical differences in characteristics between the four fluid status groups (Table 2). However, the participants were not evenly distributed in the four groups, and in the largest groups, C and D, a large number of participants reported no symptoms of either fluid overload or fluid depletion (Figure 3).

**Table 2.** Differences in baseline characteristics between groups of different types of fluid status, based on recova definitions.

Parameter	All participants (*n* = 49)		Group A (*n =* 4)		Group B (*n* = 10)		Group C (*n* = 15)		Group D (*n* = 20)		Level of significance
Age (years)	73	(67–80)	77	(69.5–81.5)	77	(74–84)	72	(59.5–75)	70.5	(61.5–78)	0.096
Clinical assessments											
Fluid overload score	0	(0–2)	2.0	(1.5–2.5)	2.0	(1–3)	0	(0–1)	0	(0–0	0.000**
Fluid depletion score	0	(0–2)	0.5	(0–1.5)	0	(0–1)	1.0	(0–2)	1.0	(1–2)	0.517
BP systolic (mmHg)	143	(121–159)	150	(123–170)	152	(149–160)	132	(119–147)	139	(122–155)	0.210
BP diastolic (mmHg)	65	(57–74)	58	(48–68)	77	(49–83)	61	(54–66)	67	(59–73.5)	0.187
Bioimpedance spectroscopy readings											
NH (kg)	73.3	(64–81.4)	78.8	(71.9–88.1)	70.8	(58.7–78)	73.1	(62.4–79.5)	73.3	(65.8–82.7)	0.704
OH pre (L)	1.7	(0.9–3.4)	0.9	(0.8–1.3)	3.8	(3.3–4.2)	2.7	(1.5–3.6)	1.0	(0.8–1.5)	0.000**
OH post (L)	0.10	(−0.8 to 1.3)	−1.55	(−2.6 to −0.35)	1.85	(1.4 to 2.1)	1.10	(0.2 to 1.4)	−0.85	(−1.6 to −0.4)	0.000**
UFV (L)	2.0	(1.3–2.7)	2.55	(1.15–3.9)	2.05	(1.1–2.4)	1.40	(1.1–2.1)	2.40	(1.7–2.7)	0.156
BMI (kg/m^2^)	27	(22.7–28.5)	28.6	(27.85–29.7)	26.2	(22–27.7)	23.9	(22.4–31.4)	27.1	(25.6–28.3)	0.360
LTI (kg/m^2^)	12	(10.6–13.6)	12.8	(9.85–14.95)	13.3	(11.5–13.9)	11.5	(10.6–12.6)	11.4	(9.7–13)	0.463
FTI (kg/m^2^)	12	(9.7–17.2)	15.6	(13.35–17.75)	10.4	(7.2–12)	10.5	(9.1–18.8)	14.7	(10.85–17.4)	0.214
Dialysis prescriptions (dialysate)											
Treatment time (hours)	4	(4–4.5)	4.3	(4–4.5)	4.0	(4–4)	4.5	(4–5)	4.0	(4–4.5)	0.356
Sessions/week	3	(3–3)	3.0	(2.5–3)	3.0	(3–3)	3.0	(3–3)	3.0	(3–3)	0.515
D-K (mmol/L)	2	(2–3)	2.5	(2–3)	2.0	(2–3)	2.0	(2–3)	2.5	(2–3)	0.922
D-Na (mmol/L)	138	(138–138)	138	(138–139)	138	(138–138)	138	(138–138)	138	(138–138)	0.437
D-temp (°C)	36.5	(36.5–36.5)	36.5	(36–36.5)	36.5	(36.5–36.5)	36.5	(36.5–36.5)	36.5	(36.5–36.5)	0.778
Target weight (kg)	73	(64.5–82)	77.5	(70–87)	71.8	(60.5–79)	75.5	(66.5–82.5)	72.8	(64.3–82.8)	0.829
Laboratory results (plasma)											
P-NT-proBNP (ng/L)	9270	(2490–19,600)	27,150	(10,735–52,400)	33,250	(17,500–41,200)	4050	(2160–10,650)	6130	(1795–11,600)	0.000**
P-CRP (mg/L)	5.9	(3.1–18)	28.0	(10.4–49.5)	13.5	(4.9–20)	7.2	(3.25–16.5)	3.95	(2.65–7.45)	0.097
P-Haemoglobin (g/L)	111	(102–117)	107	(98.5–116.5)	105	(96–11)	112	(104–117.5)	115	(104–119)	0.380
P-Na (mmol/L)	140	(138–142)	139	(137–140)	142	(140–144)	140	(138–142)	140	(138–141)	0.075
P-K (mmol/L)	4.6	(4.3–5)	4.5	(4.4–4.6)	4.6	(4.1–5)	4.3	(3.95–5.05)	4.75	(4.55–5.1)	0.269
P-Albumin (g/L)	32	(28–33)	32	(28–35)	30	(28–35)	32	(27–33)	32	(28–32.5)	0.921
P-Calcium (mmol/L)	2.22	(2.09–2.32)	2.11	(2–2.3)	2.15	(2.01–2.36)	2.25	(2.13–2.32)	2.23	(2.18–2.33)	0.473
P-Phosphate (mmol/L)	1.52	(1.05–1.85)	1.3	(0.96–1.46)	1.3	(1.05–2.24)	1.6	(0.93–1.9)	1.52	(1.21–1.73)	0.703

Differences tested for significance with kruskal–wallis *H* for non-parametric test between multiple groups.

*p < 0.05; **p < 0.01.

BP: blood pressure; BMI: body mass index; CRP: C-reactive protein; FTI: fat tissue mass index; K: potassium; LTI: lean tissue mass index; Na: sodium; NH: normal hydration weight; NT-proBNP: N-terminal pro-brain natriuretic peptide; OH: overhydration; UFV: ultrafiltration volume.

At baseline, nine individuals (18.3%) had a clinically assessed volume status score of ≥5, indicating an urgent need for target weight adjustment, and about 50% had a volume status score of 1–4. One-third of the participants had no symptoms of either fluid overload or fluid depletion. Table 3 shows the main characteristics of the whole study cohort.

**Table 3.** Baseline characteristics of all enrolled patients.

Parameter	All participants		Group A		Group B		Group C		Group D		Level of sign.
Total number	49		4		10		15		20		
Men	32	(65)	3	(75)	5	(50)	12	(80)	12	(60)	0.411
IHD	18	(37)	2	(50)	6	(60)	6	(40)	4	(20)	0.164
CHF	23	(47)	2	(50)	2	(50)	6	(40)	10	(50)	0.937
Stroke/TIA	10	(20)	3	(75)	2	(20)	2	(13)	3	(15)	0.043
PVD	10	(20)	2	(50)	1	(10)	3	(20)	4	(20)	0.419
Hypertension	46	(94)	4	(100)	8	(80)	15	(100)	19	(95)	0.201
DM type 1	4	(8)	0		0		2	(13)	2	(10)	0.600
DM type 2	16	(33)	2	(50)	10	(40)	6	(40)	4	(20)	0.455
Total symptom score, thresholds and trigger											0.167
Level 0 points	16	(33)	0		0		7	(46)	9	(45)	
Level 1–4 points	24	(49)	3	(75)	7	(70)	6	(40)	8	(40)	
Level 5–6 points	8	(16)	1	(25)	3	(30)	1	(7)	3	(15)	
Level ≥7 points	1	(2)	0		0		1	(7)	0		

Differences tested for significance with the chi-square test.

Data presented as numbers of participants (%).

*p < 0.05; **p < 0.01.

IHD: ischaemic heart disease; CHF: congestive heart failure; DM: diabetes mellitus; PVD: peripheral vascular disease; TIA: transient ischaemic attack.

### Intervention evolution over time

Prior to the intervention, bioimpedance measurements were performed 0.5 times/patient/month, and there was no significant difference in target weight adjustment frequency between the two haemodialysis units. In the first unit, the staff were given the full responsibility to follow the protocol without further support. However, only 67% of the expected assessments were performed. Therefore, in the second unit, the first author visited the dialysis unit every second week to check if the intervention was carried out as intended and to support the nurses in their response to recognized fluid alterations. Hence, in the second haemodialysis unit, 100% of the expected assessments were performed.

### Process measures

The monthly frequencies of both performed bioimpedance measurements and target weight adjustments increased by 1.5 in the first unit. In the second unit, there was a twofold increase in bioimpedance measurement frequency and a close to twofold increase in target weight adjustment frequency (Table 4). There was a correlation between frequency of bioimpedance measurements and frequency of target weight adjustments. Fluid overload symptoms correlated with OH post and NT-proBNP (Table 5).

**Table 4.** Measure of process.

Parameter	Baseline		Follow-up			Level of significance
Haemodialysis unit 1 (*n* = 27)						
BIS/month	0.50	(0.50–0.67)	0.75	(0.75–1.0)		0.035
TW adjustments/month	0.50	(0.33–0.83)	0.75	(0.50–1.25)		0.021
Haemodialysis unit 2 (*n* = 22)						
BIS/month	0.50	(0.50–0.67)	1.00	(0.67–1.0)		0.002**
TW adjustments/month	0.67	(0.33–0.83)	1.00	(0.33–1.33)		0.005**

Significance of differences between baseline and follow-up tested with wilcoxon’s non-parametric test between dependent groups.

*p < 0.05; **p < 0.01.

BIS: bioimpedance spectroscopy; TW: target weight.

**Table 5.** Correlations in entire sample.

	FD score	OH pre	OH post	NT-proBNP	BIS/month	TW changes/month
FO score	0.24	0.17	0.29	0.36	0.24	0.22
FD score		−0.15	−0.08	−0.19	0.15	0.20
OH pre (L)			0.76	0.36	−0.01	−0.03
OH post (L)				0.21	−0.05	−0.05
NT-proBNP (ng/L)					0.20	0.09
BIS/month						0.63

*p < 0.05; **p < 0.01.

BIS: bioimpedance; FD: fluid depletion; FO: fluid overload; NT-proBNP: N-terminal pro-brain natriuretic peptide; OH: overhydration; TW: target weight.

### Outcome measures

#### Group A – symptoms of fluid overload but negative OH

**Figure 2.**
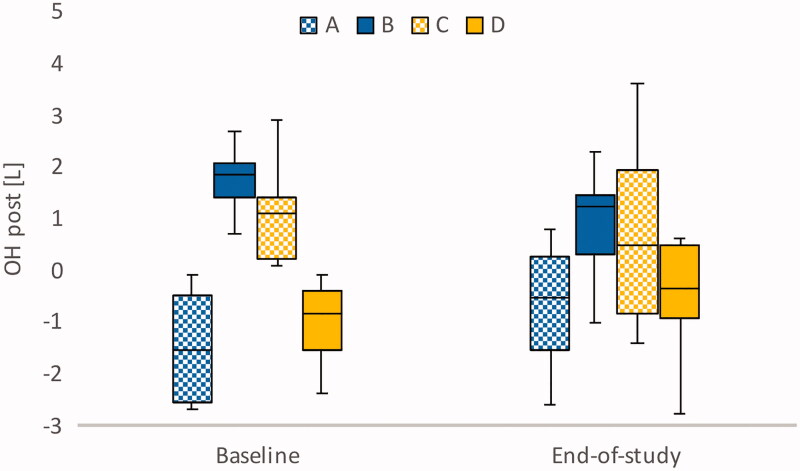
Bioimpedance-measured median overhydration (OH) post in the four groups at baseline and at end-of-study.

**Figure 3.**
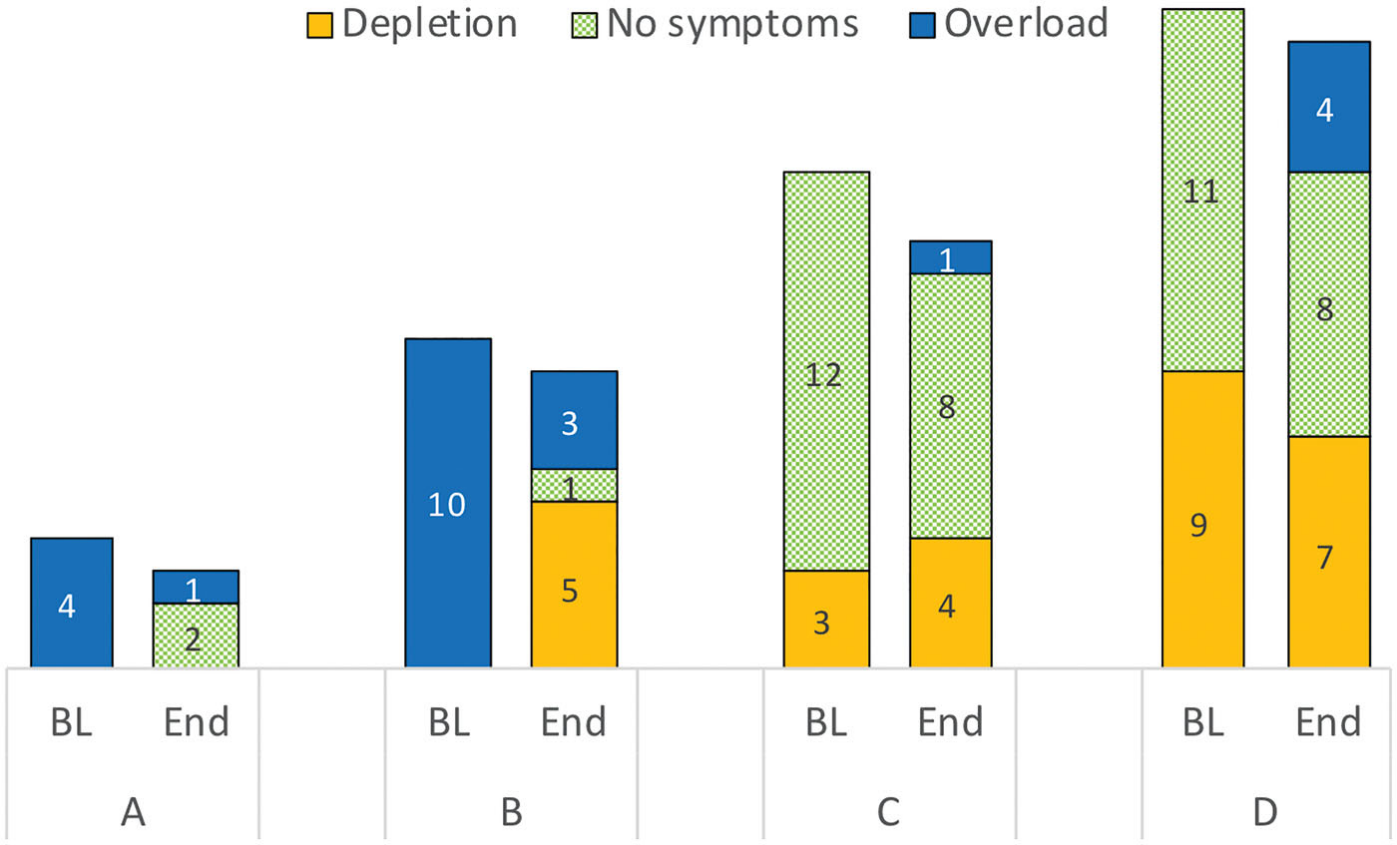
Comparison of distribution of pre-dialysis fluid symptom status in each group (frequencies), at baseline (BL) and at end-of-study (End).

In group A (*n* = 4), OH post was −1.55 (−2.6 to −0.35) L at baseline, despite symptoms of fluid overload (Figure 2). However, in contrast to suggested clinical response (Table 1), the prescribed target weight increased in three cases. Despite this, two participants were relieved from symptoms of fluid overload (Figure 3), and at the end of the study all participants had reached the Recova-defined target for group A, that is OH post below 0 L. At baseline, NT-proBNP was 27,150 (10,735–52,400) ng/L. The levels decreased in three cases but increased in one, where the participant had a decrease in lean and adipose tissue. At the group level, there was no significant change in NT-proBNP (Table 6).

**Table 6.** Differences between baseline and follow-up observations in subgroups, significance tested with Wilcoxon’s non-parametric test between dependent groups.

Parameter	Baseline		End-of-study		Significance
Group A: Fluid overload symptoms but negative OH (*n* = 4)					
Fluid overload score	2	(1.5–2.5)	0	(0–0.5)	0.102
Fluid depletion score	0.5	(0–1.5)	0	(0–0)	0.317
OH pre (L)	0.9	(0.8–1.3)	1.45	(0.85–2.1)	0.144
OH post (L)	−1.55	(−2.6 to −0.35)	−0.70	(−1.9 to −0.15)	0.715
NT-proBNP (ng/L)	27,150	(10,735–52,400)	30,950	(9851–54,650)	0.715
BP systolic (mmHg)	150	(123–170)	166	(131–194)	0.068
BP diastolic (mmHg)	58	(48–68)	60	(55–76)	0.144
Target weight (kg)	77.5	(70–87)	77.5	(72.8–87.5)	0.197
UFV (L)	2.55	(1.2–3.9)	2.70	(1.6–3.5)	1.000
BIS/month	0.42	(0.33–0.67)	0.75	(0.75–0.88)	0.141
DW adjustments/month	0.67	(0.25–0.92)	0.88	(0.50–1.38)	0.197
Group B: Fluid overload symptoms and positive OH (*n* = 10)					
Fluid overload score	2	(1–3)	0	(0–1)	0.033
Fluid depletion score	0	(0–1)	0	(0–1)	0.129
OH pre (L)	3.8	(3.2–4.2)	2.9	(2.6–3.4)	0.047
OH post (L)	1.85	(1.4–2.1)	1.25	(0.3–1.5)	0.074
NT-proBNP (ng/L)	33,250	(17,500–41,200)	30,150	(22,700–57,700)	0.953
BP systolic (mmHg)	152	(149–160)	156	(126–172)	0.959
BP diastolic (mmHg)	77	(49–83)	71	(48–75)	0.314
Target weight (kg)	71.8	(60.5–79)	70.3	(56.5–79)	0.067
UFV (L)	2.05	(1.1–2.4)	1.85	(1.3–3.1)	0.919
BIS/month	0.67	(0.42–0.75)	1.0	(0.75–1.0)	0.176
DW adjustments/month	0.67	(0.50–0.75)	1.0	(0.63–1.33)	0.400
Group C: Fluid depletion or no symptoms but positive OH (*n* = 15)					
Fluid overload score	0	(0–1.0)	0	(0–1.0)	0.931
Fluid depletion score	1	(0–2.0)	1	(0–2.0)	0.526
OH pre (L)	2.7	(1.5–3.6)	2.7	(1.6–4.5)	0.826
OH post (L)	1.10	(0.2 to 1.4)	1.20	(−0.9 to 2.7)	0.410
NT-proBNP (ng/L)	4050	(2160–10,650)	3320	(2430–9820)	0.256
BP systolic (mmHg)	132	(119–147)	130	(122–152)	0.513
BP diastolic (mmHg)	61	(54–66)	60	(56–69)	0.132
Target weight (kg)	75.5	(66.8–82.5)	75.5	(66.5–82.3)	0.503
UFV (L)	1.4	(1.1–2.1)	2.1	(1.3–2.4)	0.140
BIS/month	0.5	(0.33–0.50)	0.67	(0.50–1.0)	0.005
DW adjustments/month	0.5	(0.17–0.67)	0.5	(0.25–1.17)	0.049
Group D: Fluid depletion or no symptoms and negative OH (*n* = 20)					
Fluid overload score	0	(0–0)	0	(0–1.5)	0.135
Fluid depletion score	1	(0–3)	1	(0–2)	0.340
OH pre (L)	1.0	(0.8–1.5)	1.3	(0.7–2.1)	0.248
OH post (L)	−0.85	(−1.6 to −0.4)	−0.5	(−1.0 to 0.1)	0.057
NT-proBNP (ng/L)	6130	(1795–11,600)	9625	(2070–21,700)	0.033
BP systolic (mmHg)	139	(122–155)	151	(123–165)	0.057
BP diastolic (mmHg)	67	(59–74)	68	(58–74)	0.852
Target weight (kg)	72.8	64.3–82.8)	73.4	(66–84.5)	0.024
UFV (L)	2.4	(1.7–2.7)	1.9	(1.45–2.9)	0.313
BIS/month	0.67	(0.5–0.75)	0.75	(0.71–1.0)	0.064
DW adjustments/month	0.5	(0.33–0.83)	0.75	(0.59–1.33)	0.005

*p < 0.05; **p < 0.01.

BIS: bioimpedance spectroscopy; BP: blood pressure; FO: fluid overload; NT-proBNP: N-terminal pro-brain natriuretic peptide; OH: overhydration; UFV: ultrafiltration volume.

#### Group B – symptoms of fluid overload and positive OH

At baseline, all participants in group B (*n* = 10) had symptoms of fluid overload, and OH post was 1.85 (1.4–2.1) L. The target weights decreased in seven cases, were unchanged in two, and increased in one. At the end of the study, five individuals had reached the target for group B, that is OH post ±1.1 L. Three participants had remaining symptoms of fluid overload, one was relieved of symptoms, and five had symptoms of fluid depletion. At baseline, this group had the highest NT-proBNP, 33,250 (17,500–41,200) ng/L, and although median pre-dialysis OH decreased from 3.8 to 2.9 L (*P* = 0.047) NT-proBNP was not affected.

#### Group C – symptoms of fluid depletion or absence of symptoms but positive OH

In group C (*n* = 15), OH post was 1.10 (0.2–1.4) L at baseline. The vast majority (12 out of 15 individuals) had no symptoms of either fluid overload or fluid depletion. The target weights increased in six cases, decreased in six, and were unchanged in three. At the end of the study, five individuals had reached the target for group C, that is OH post 0–2 kg. Group C had the lowest median NT-proBNP, 4050 (2160–10,650) ng/L, and NT-proBNP did not change on a group level.

#### Group D – symptoms of fluid depletion or absence of symptoms and negative OH

In group D (*n* = 20), nine participants had symptoms of fluid depletion. Eleven participants reported no symptoms. At baseline, OH post was −0.85 (−1.6 to −0.4) L. At the end of the study, the target weight had increased in 13 cases. The target for group D, that is OH post ±1.1 L, was reached in 15 cases. When the target weight increased from 72.8 to 73.4 kg (*P* = 0.024), OH post increased to −0.5 (−1.0 to 0.5) L. At the end of the study, NT-proBNP had increased from 6130 to 9625 ng/L (*P* = 0.033).

### Contextual elements that interacted with the intervention

At the first haemodialysis unit, the intervention ran for 4 months, May–August 2019. This coincided with summer holidays and staff vacations, which probably affected adherence to the study protocol and, hence, process measures.

In both haemodialysis units, changes in participants’ body composition were found to interact with outcome measures. Due to changes in adipose and lean tissue mass, the target weight, as estimated at baseline, was not always adequate at the end of the study. For example, the suggested clinical response according to Recova is to either decrease (groups A and B) or increase (groups C and D) the target weight in order to bring relief from symptoms (Table 1). However, in group A, the target weight decreased in one participant only. Still, all four participants reached the target, OH post below 0 L, and two out of four individuals no longer had symptoms of fluid overload. This was due to an increase in lean and adipose tissue. Conversely, in one case in group D, although the staff members adhered to the intervention and increased the target weight from 83.5 to 87.5 kg, the participant had symptoms of fluid depletion and a negative OH post because of an increase in lean and adipose tissue corresponding to 4.9 kg. A third example is in group C; due to severe decline in body weight, one participant developed symptoms of fluid overload despite a 10 kg target weight reduction.

### Details about missing data

End-of-study data on fluid symptoms were missing in five cases. At baseline, two of these participants had reported fluid overload, whereas three had reported absence of symptoms. All five were from the first haemodialysis unit.

## Discussion

This prospective intervention study evaluated the effect of a decision aid for recognition and correction of volume alterations in haemodialysis patients, the Recova tool, which combines clinical assessment and bioimpedance in target weight determination. One of the most important findings of the study is the discordance between clinically assessed fluid status and hydration status as measured using bioimpedance. Out of 49 enrolled haemodialysis patients, from two haemodialysis units, 18% (*n* = 9) were found to have symptoms indicating an urgent need for target weight adjustment.

Based on the participants’ clinically assessed symptoms and their hydration status as measured using bioimpedance, four fluid status groups (A, B, C, and D) were distinguished, and, by the end of the study, the frequencies of bioimpedance measurements and target weight adjustments had increased. A majority of the participants with both fluid overload symptoms and positive OH (group B), had decreased target weight, fewer symptoms, and decreased pre-dialysis OH. In the group of participants with symptoms of fluid depletion and negative OH post (group D), the target weight had increased in 13 out of 20 participants. In the two groups in which clinical assessments and bioimpedance measurements were in conflict (group A and group C), the intervention had no effect at a group level.

Only four study participants were in group A, corresponding to symptoms of fluid overload but negative OH post. According to Recova, the appropriate clinical response to this group would be a decrease of target weight for symptom reduction, despite negative OH post. However, the target weight did not change significantly. Conversely, median OH post increased, and our results indicate that staff were guided more by the bioimpedance device, trying to get to OH post = 0 in all cases, than by the protocol. However, the low number of participants in the group prevents generalizability, and moreover the increase in target weight may be due to contextual elements affecting the outcome, that is individuals’ increase in lean and adipose tissue. Nevertheless, because our results could be interpreted as indicating that staff members needed more training to gain deeper understanding of the relevant applications of bioimpedance, we want to stress the importance of individualized fluid management in haemodialysis. Since fluid depletion post-dialysis is associated with a survival benefit (23), a target weight of 1–2 kg below normohydration weight may be appropriate in some subjects.

Group B, with symptoms of fluid overload and positive OH post, had the highest NT-proBNP. Interestingly, both group A and group B – with participants reporting fluid overload symptoms – had significantly higher NT-proBNP than groups C and D. This is despite group A having negative OH post and group B having positive OH post. This finding underlines the importance of combining bioimpedance with other measures of fluid status for individualized target weight determination (12,13). In group B, the target weight had decreased by the end of the study, as recommended by Recova. As high OH in combination with high NT-proBNP is associated with increased mortality (24), the improved hydration status in group B may be one of the most important effects of this implementation intervention. When pre-dialytic OH decreased, the number of participants reporting symptoms of fluid depletion also increased. Intra- and post-dialytic complications can make fluid removal difficult even in patients with significant fluid overload (25). However, symptoms of fluid depletion, as reported in group B after the intervention, may be related to the use of anti-hypertensive medication and to dialysis prescription rather than fluid depletion per se (26). For patients to achieve an adequate target weight, without experiencing increased intradialytic fluid depletion symptoms, a different dialysis schedule – for example more frequent or longer dialysis sessions – may be required.

According to Recova, individuals with positive OH but symptoms of fluid depletion, group C, may benefit from a target weight 1–2 kg above normohydration. Correction to a bioimpedance measured OH = 0 may cause hypotension if the observed OH is in combination with malnutrition, inflammation, low BMI, high age, and/or malignancy. There is no evidence that attaining euvolemia is feasible or desirable under these circumstances (27–29). However, in our study sample, participants in group C did not have lower albumin, higher CRP, lower BMI, or higher age than participants in the other groups (Table 2). Still, the relatively low NT-proBNP (significant) and blood pressure (non-significant) confirm that individuals in group C may tolerate an increased target weight despite positive OH. On the other hand, the vast majority of individuals in group C had no symptoms of either fluid overload or fluid depletion (Figure 3). The observed absence of significant difference in target weight at the end of the study may thus be clinically appropriate.

In our study sample, a large proportion of individuals presented symptoms of fluid depletion and negative OH post, group D. In a recent trial, normalization of volume status in patients with negative OH resulted in a significant reduction in intradialytic hypotension (30). In our study, the target weight increased in this group, as recommended by Recova, and there was a small decrease in the number of individuals with fluid depletion symptoms. However, NT-proBNP also increased, with 50%. This parameter was not investigated in the study by Patel et al. (30). The increase in NT-proBNP after target weight increase raises some concern, as elevated NT-proBNP is associated with increased mortality in haemodialysis patients (18). However, the use of NT-proBNP as a marker of fluid overload in haemodialysis is controversial (31). The vast majority of haemodialysis patients have elevated NT-proBNP levels as the peptide is renally excreted. In addition, a large proportion of the study participants had ischaemic heart disease and/or congestive heart failure (Table 3). It has been argued that serial NT-proBNP levels need to be doubled or halved in haemodialysis patients to confidently exclude changes due to analytical and biological variation alone (32).

Four individuals in group D reported symptoms of fluid overload at the end of the study. One of these individuals had reported muscle cramps as the only clinical symptom at baseline but reported a sudden increase in fluid overload symptoms between the eighth and the ninth (last) assessment, from 1 to 8 points in 3 weeks. In this case NT-proBNP also increased from 6700 ng/L to above 70,000 ng/L at the end of study. This remarkable increase probably made a substantial contribution to the increased NT-proBNP level in group D.

Accurate assessment of fluid volume status remains a concern in haemodialysis. Clinical assessment of fluid status may be imprecise and subjective (3), and there is still no consensus on which target to aim for in maintenance haemodialysis patients (29). Our results also suggest an individual’s fluid status may change rapidly. Bioimpedance technology may add valuable information to this complex decision-making process. However, under certain conditions there may be a discrepancy between bioimpedance measurements and clinical assessment. Hence, bioimpedance should not be used in isolation, but in combination with clinical assessment (12). Recova defines four different types of fluid status groups and provides an overview of patients’ related conditions that should be taken into consideration when using bioimpedance in target weight determination. It also systematizes the process of clinical fluid status assessment (17). The purpose of the decision aid is to facilitate interprofessional communication by defining when and how the target weight should be evaluated.

### Limitations

This interventional study has several limitations. Firstly, the study is based on a relatively small sample, and the participants were not evenly distributed when divided into groups. This affects the precision and accuracy of our interpretations and hence decreases the generalizability of our findings. Moreover, we realize that the results of the intervention could have been made clearer if a ‘normal’ group had been identified at baseline – that is, participants without symptoms and bioimpedance-measured hydration status within the normal range, −1.1 L to +1.1 L, who were now included in groups C and D (Table 1) although not requiring a target weight adjustment.

In order to prevent symptoms of fluid depletion when the target weight is decreased, it is usually necessary to gradually and continuously adjust blood pressure medication, alter dialysis prescriptions, and provide dietary counselling on sodium reduction. In Recova, it is suggested that target weight reduction should not be reinforced rapidly (17). However, in this study these measures were not taken into consideration, but in further research, evaluating the Recova tool, we recommend they are. Also, Recova emphasizes the need for preservation of residual renal function, but this was not routinely measured at the clinic where the trial was conducted. The nurses were encouraged to discuss residual renal function with the study participants, but this parameter could not be included in the analysis. To compensate for this limitation, all patients with residual renal function large enough to negate the need for ultrafiltration were excluded from the study.

One strength of Recova is the potential for a multidisciplinary approach. However, this implementation intervention primarily addressed nurses. The physicians at the clinic received only brief information about the tool. It is possible that a multi-professional approach could have improved adherence to protocol and the effect of the implementation. The implementation intervention was introduced similarly in two haemodialysis units, but in the first unit only 67% of the expected assessments were performed. In the second unit, the intervention was more closely monitored, and 100% of the expected assessments were performed, and there was a greater increase in frequencies of bioimpedance measurements and target weight adjustments. This highlights both the importance of having well-established routines for target weight assessments (15) and the need for tailored implementation strategies.

## Conclusions

This prospective intervention study evaluated the effect of implementing a decision aid, Recova, in haemodialysis care. After the implementation, the monthly frequencies of bioimpedance measurements and target weight adjustments increased, and individuals with fluid overload symptoms and positive OH post improved in symptoms and hydration status. Study participants with symptoms of fluid depletion and negative OH had increased target weights, as proposed. Using Recova in guiding fluid management in haemodialysis may be beneficial. However, monitoring and adherence seem essential. This study is based on a small sample, and further studies are required to confirm the generalizability of our findings and the effect on patient outcome.

## Appendix 1 Appendix A. The Recova^®^ thresholds and triggers for action.

Based on the total score as given in the Recova symptom scoring system, see Figure 1.
